# Human Periodontal Ligament Stem Cell-Derived Exosomes Promote Bone Regeneration by Altering MicroRNA Profiles

**DOI:** 10.1155/2020/8852307

**Published:** 2020-11-17

**Authors:** Ting Liu, Wenyun Hu, Xue Zou, Jingchen Xu, Shushu He, Le Chang, Xinyi Li, Yuanyuan Yin, Mi Tian, Ziyu Li, Jialiang Zhou, Xiaoge Jiang, Song Chen

**Affiliations:** ^1^State Key Laboratory of Oral Diseases, National Clinical Research Center for Oral Diseases, Department of Orthodontics, West China Hospital of Stomatology, Sichuan University, Chengdu, Sichuan 610041, China; ^2^Department of Medical Technology, Gannan Healthcare Vocational College, Ganzhou 341000, China; ^3^Clinical Research Center, The Affiliated Hospital of Guizhou Medical University, Guiyang 550004, China

## Abstract

The role and underlying mechanism of exosomes derived from human periodontal ligament stem cells (PDLSC) in osteogenesis are unclear. In the present study, we identified the exosomes derived from PDLSCs and found that osteogenic induction can enhance the osteogenic ability of PDLSC-derived exosomes in promoting the osteogenic differentiation of rat bone marrow stem cells (BMSCs). To investigate the underlying mechanism, we analyzed the exosomal miRNA expression profiles of undifferentiated and osteogenic differentiated PDLSCs by RNA sequencing. The results showed that seventy-two miRNAs were upregulated and thirty-five miRNAs were downregulated after osteogenic induction. The results of Gene Ontology analysis and pathway analysis demonstrated that the target genes of differentially expressed exosomal miRNAs participate in the regulation of a variety of biological processes, such as catalytic activity, protein binding, metabolic processes, cell development, and differentiation, and are enriched in osteogenic differentiation-related pathways, such as MAPK signaling, AMPK signaling, and insulin signaling pathways. Our results reveal for the first time that the exosomal miRNAs derived from osteogenic differentiated PDLSCs may promote the osteogenic differentiation of BMSCs, which provides a basis for further research on the regulatory function of exosomal miRNA of PDLSCs during osteogenesis.

## 1. Introduction

Bone defects can seriously damage the patient's aesthetics and musculoskeletal function. In the past few decades, bone engineering based on mesenchymal stem cells (MSCs) is considered a promising strategy as MSCs exhibit the capacity to differentiate into multiple lineage cells *in vitro*. Dental stem cells (DSCs) have been widely accepted as derived from the neural crest and are becoming extremely important in tissue engineering [1]. DSCs have shown amazing therapeutic abilities in the treatment of oral-facial, cornea, cardiovascular, hepatic, and autoimmune diseases [2–4]. Human periodontal ligament stem cells (hPDLSCs) were MSCs isolated from the human periodontal ligament which is a soft connective tissue located between the tooth root and the alveolar socket. hPDLSC was reported to have the best regeneration capability and multipotency over other DSCs, such as dental pulp stem cells and dental follicle progenitor cells [5]. It presents an easily accessible source of MSCs and has been used for bone regeneration [6].

Recent reports have indicated that the paracrine pathway might be the principal mechanism by which MSCs contribute to tissue regeneration [7, 8]. Extracellular vesicles (EVs), 30–150 nm-sized membranous vesicles released by cells, have attracted attention as cell-to-cell communication molecules. EVs can transfer RNA, noncoding RNA, and proteins. They can regulate the repair and regeneration process of the injured site by affecting the proliferation, migration, differentiation, and immune environment of the recipient cell [9, 10]. It has been reported that PDLSC-derived exosomes play a vital role in the process of enhancing nerve repair [11, 12], promoting fracture healing, osteochondral regeneration, and repair of skull defects [8, 13–15].

The biological effects of exosomes largely depend on the cellular origin and physiological status of donor cells [16]. Studies have revealed that exosomes derived from osteogenic differentiation-induced and nonosteogenic-induced mesenchymal stem cells such as adipose stem cells (ADSCs) and BMSCs have different abilities in osteogenesis differentiation. Exosomes derived from stem cells induced by osteogenic differentiation have a more substantial osteogenic effect [17, 18]. The studies also indicated that exosomes derived from osteogenic-induced differentiation and nonosteogenic-induced differentiation stem cells contain different microRNAs, which may be responsible for the difference in osteogenic effects [17, 19]. MicroRNA is a kind of endogenous noncoding RNA, which can act as a negative regulator of posttranscriptional gene expression by combining with the 5′end “seed” region or the 3′untranslated region (UTR) of the target mRNAs [20]. Exosomes can regulate epigenetic processes by delivering miRNAs to recipient cells and regulate the biological function of recipient cells in bone regeneration [21, 22]. However, the role of PDLSC-derived exosomes in bone regeneration and their exosomal miRNA profile remain unknown.

In this study, we revealed the osteoinductive property of exosomes derived from PDLSCs at different stages during osteoinduction process. Besides, we compared the overall miRNA expression in the exosomes derived from PDLSCs before and after osteogenic differentiation and conducted bioinformatics analyses. Our study may provide a basis for further research on the osteogenic regulatory function of exosomal miRNAs of PDLSCs.

## 2. Materials and Methods

This study was approved by the Ethical Committee of the West China Stomatology Hospital of China Sichuan University, Sichuan, China.

### 2.1. Cell Isolation and Identification

#### 2.1.1. Cell Isolation

Human PDLSCs were isolated from healthy PDL tissues of premolars extracted from 10 patients aged 12-18 years undergoing orthodontic treatment. Primary PDLSCs were separated and expanded as described previously [23]. Briefly, PDL tissues were gently removed from the root surface and digested with 3 mg/mL collagenase type 1 (Sigma-Aldrich, USA) and 4 mg/mL collagenase type 2 (Roth, USA) for 30 min at 37°C. The cells were seeded into 25 cm^2^ culture flasks (Corning, Lowell, MA, USA) with *α*-minimum essential medium (*α*-MEM, Gibco, USA) supplemented with 10% fetal bovine serum (FBS, Gibco, BRL, USA) and 1% penicillin-streptomycin solution (Gibco, USA) and then incubated at 37°C with 5% CO2. PDLSCs at passages three to five were used for experiments. Primary BMSCs were obtained as described previously [24]. In brief, the bone marrow was flushed out from the femurs of 14-day-old Sprague-Dawley (SD) rats with Dulbecco's modified Eagle's medium (DMEM, Gibco, USA) supplemented with 10% FBS (Gibco, BRL, USA) and 1% penicillin-streptomycin solution (Gibco, USA). After being centrifuged at 1000 rpm for 5 min, the cells were cultured in 25 cm^2^ culture flasks (Corning, Lowell, MA, USA) at 37°C with 5% CO_2_. BMSCs at passage three were used in the following experiments.

#### 2.1.2. Cell Identification

Approximately 1 × 10^6^ PDLSCs at the third passage were added in blocking buffer and incubated with anti-STRO-1 immunoglobulin (Ig)M (Santa Cruz, CA, USA) for 1 h at 37°C, then incubated with goat anti-mouse IgM FITC secondary antibodies (Molecular Probes, Eugene, Carlsbad, CA, USA) for an additional 1 h. PDLSCs incubated with the secondary antibody only was used as a negative control. Another 1 × 10^6^ PDLSC was incubated with anti-CD146-PE, anti-CD45-FITC, and anti-CD90-APC antibodies (BD Biosciences, USA), at 37°C for 30 min. 1 × 10^6^ BMSCs at the third passage were incubated with antibodies at 37°C for 30 min, including anti-CD29-FITC, anti-CD 45-FITC, anti-CD44-FITC, anti-CD11b/c-FITC, and anti-CD90-FITC (BioLegend, CA, USA). The cells were then analyzed by flow cytometry (Attune NxT, Invitrogen, USA) and Treestar FlowJo software.

### 2.2. Exosome Isolation and Characterization

#### 2.2.1. Conditioned Medium Collection

PDLSCs were cultured in exosome-depleted medium for collection of conditioned medium [25]. The cells were treated with osteogenic-inducing medium (OM, culture media supplemented with 10 mM *β*-glycerophosphate, 50 *μ*M ascorbic acid, and100 nM dexamethasone) for 3, 7, and 14 days, and the conditioned medium was collected for extraction of exosomes after incubating for an additional 48 h. Besides, in order to further investigate the role of exosomes in osteogenesis, PDLSCs after 14 days of osteoinduction were washed with PBS and cultured in exosome-depleted culture media with or without the neutral sphingomyelinase (N-SMase) inhibitor GW4869 (10 *μ*M) for another 24 h. The same amount of dimethyl sulfoxide (DMSO) was added in the control group. The conditioned medium with or without GW4869 was prepared into osteogenic medium for BMSC culture, termed as CM+GW4869 and CM.

#### 2.2.2. Isolation of Exosomes

A series of differential centrifugation (300 × g for 10 min, 2000 × g for 20 min, 10000 × g for 30 min) was conducted with the conditioned medium containing exosomes derived from undifferentiated PDLSCs (Exos_NC) and exosomes derived from PDLSCs after 3, 7, and 14 days of osteogenic induction (Exos_D3, Exos_D7, Exos_D14). The supernatant was filtrated through a 0.22 *μ*m filter. Exosomes were then extracted by ultracentrifugation (Beckman 70Ti ultra rotor, USA) at 100000 × g for 70 min. The exosomes were resuspended in PBS and stored at -80°C.

#### 2.2.3. Transmission Electron Microscopy (TEM)

TEM was used to observe the morphology of exosomes. 20 *μ*L of the exosomes was dropped onto formvar carbon-coated copper grids for 1 minute, and excess suspension liquid was removed by filter paper. Then, the grids were stained with 2% phosphotungstic acid for 5 minutes at room temperature, followed by fixing with 2% glutaraldehyde for 5 minutes. Images were captured under 80 kV with a transmission electron microscope (JEM-2100; Jeol Ltd., Tokyo, Japan) [26].

#### 2.2.4. Nanoparticle Tracking Analysis (NTA)

The particle size and particle concentration of exosomes were measured by using NTA. The exosomes were diluted with PBS and analyzed using the ZetaView PMX 110 (Particle Metrix, Germany). The results were analyzed with NTA analytical software (ZetaView 8.04.02 SP2).

#### 2.2.5. Western Blotting

The cells and exosomes were lysed in RIPA lysis buffer (Beyotime, China), and protein content was quantified using a BCA Protein Assay Kit (Beyotime Biotechnology, China). Western blotting was conducted as described previously [27]. Briefly, an equal amount of proteins from cells or exosomes was loaded on 10% gels and separated by SDS-PAGE and transferred to PVDF membranes (Millipore, Bedford, MA, USA). The membranes were blocked with 5% evaporated skimmed milk and then incubated with each primary antibody, including anti-CD63, anti-TSG101, and anti-calnexin (Abcam, USA, 1 : 1000 dilution) at 4°C overnight. The respective secondary antibodies (Cell Signaling Technology, USA, 1 : 5000 dilution) were incubated for 1 hour at room temperature. The membrane was washed three times with TBST after each incubation, An ECL kit (Biotech) was used to visualize the protein bands.

### 2.3. Exosome Uptake Assay

Exosomes and PKH-26 dye (Sigma-Aldrich, USA) were incubated together in Diluent C. After 5 minutes, DMEM containing 5% FBS was added to stop staining. Then, the exosomes were washed in PBS at 100000 × g for 1 h. After that, the exosomes were added to the BMSC culture medium. After 6 hours of incubation, the cells were fixed with 4% paraformaldehyde for 10 min. The cytoplasm of BMSCs was stained with 5-(and 6)-carboxyfluorescein diacetate succinimidyl ester (CFDA-SE) solution according to the manufacturer's instructions. The cell nuclei were stained with 6-diamidino-2-phenylindole (DAPI) solution. An LSM710 confocal imaging system (Carl Zeiss, Oberkochen, Germany) was used to capture the image [17].

### 2.4. Alkaline Phosphatase (ALP) Activity Assay and ALP Staining

BMSCs were lysed by cell lysis buffer (Beyotime, China) on day 7. ALP assay was performed using an Alkaline Phosphatase Assay Kit (Beyotime, China), and optical density (OD) values were determined using a spectrophotometer (Varioskan LUX, Thermo, USA) at 405 nm. NBT/BCIP staining kit (Beyotime Biotechnology, China) was used to obtain ALP staining on day 7, as described in detail previously [28].

### 2.5. Alizarin Red S (ARS) Assay

ARS assay was conducted on day 14. After being fixed for 20 min with 1 mL 4% neutral formaldehyde solution, the wells were washed with PBS, and 1 mL 1% alizarin red S (Sigma-Aldrich) was added in each well for 5 min. The plate was washed three times with PBS. To quantify the calcification, alizarin red was dissolved in 100 mmol/L cetylpyridinium chloride for 30 minutes and evaluated by absorbance values at 562 nm.

### 2.6. Real-Time Quantitative Reverse Transcription-Polymerase Chain Reaction (qRT-PCR)

Total RNAs were extracted by using TRIzol™ Reagent (Invitrogen, USA) from cells, and cDNA was synthesized by PrimeScript™ RT Master Mix (TAKARA, Japan). RNA of exosomes was extracted by the exoRNeasy Serum/Plasma Maxi Kit (Qiagen, USA). miRNA reverse transcription was performed by miRNA First-Strand cDNA Synthesis Tailing Reaction Kit (B532451, Sangon Biotech, Shanghai, China). Quantitative polymerase chain reaction was performed using SYBR Premix Ex Taq™ II (TAKARA, Japan). The expression of target genes including Runt-related transcription factor 2 (*RUNX2*), alkaline phosphatase (*ALP*), Osterix, *GAPDH*, miR-122-5p, miR-142-5p, miR-25-3p, miR-192-5p, let-7b-5p, miR-100-5p, and miR-125b-5p was detected. Sequences are showed Table 1. GAPDH and U6 were used as internal controls for mRNA and miRNA analyses, respectively.

### 2.7. RNA Sequencing

Exosomal RNA was isolated by the exoRNeasy Serum/Plasma Maxi Kit (Qiagen, USA). RNA concentration was measured using a Qubit® RNA Assay Kit in a Qubit® 2.0 Fluorometer (Life Technologies, CA, USA), and the RNA integrity was assessed using an RNA Nano 6000 Assay Kit of the Agilent Bioanalyzer 2100 system (Agilent Technologies, CA, USA). For small RNA libraries, 3 *μ*g RNA per sample was used to generate a small RNA library for the analysis of miRNAs. Sequencing libraries were generated using NEBNext® Multiplex Small RNA Library Prep Set for Illumina (NEB). The libraries obtained from different samples were performed on an Illumina Hiseq 2500 platform and verified using three parallel replicates. Bowtie [29] was used to map small RNA tags to the reference sequences. Taking miRBase20.0 as a reference, miRDeep2 [30] and sRNA-tools-cli software were used in looking for potential known miRNA. miREvo [31] and miRDeep2 software were integrated to predict novel miRNAs.

### 2.8. Bioinformatics Analysis

Differential expression analysis of the two groups was performed using the DESeq R package (1.8.3) with thresholds of corrected *p* values < 0.05 and foldchange(FC) ≥ 2. Gene Ontology (GO) enrichment analysis was used on the target gene candidates of differentially expressed miRNAs [32]. DAVID Bioinformatics Resources 6.8 (https://david.ncifcrf.gov/) and KOBAS software [32] were used to test the statistical enrichment of the target gene candidates in the Kyoto Encyclopedia of Genes and Genomes (KEGG) pathway. The target gene of ten miRNAs were predicted by three online analysis platforms: miRWalk (http://mirwalk.umm.uni-heidelberg.de/), miRDB (http://mirdb.org/), and TargetScan (http://www.targetscan.org). The results were analyzed using jvenn (http://jvenn.toulouse.inra.fr/app/example.html). The miRNA-mRNA network was conducted using Cytoscape 3.60.

### 2.9. Statistical Analysis

SPSS 17.0 software was used for statistical analysis. Student's *t*-tests were used for comparisons between two groups. Comparisons among three or more groups were analyzed by one-way analysis of variance followed by Tukey's post hoc test. For all data, *p* values < 0.05 were considered statistically significant. Each experiment was performed three times.

## 3. Results

### 3.1. Characteristics of Human PDLSCs and Rat BMSCs

**Figure 1:**
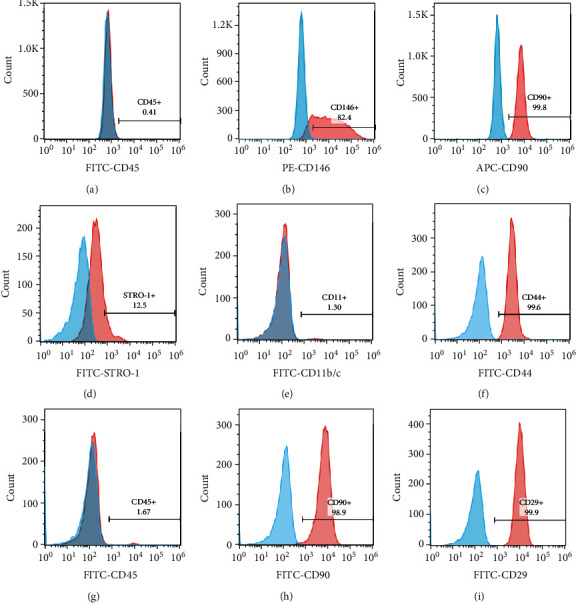
Identification of hPDLSCs and rBMSCs: (a–d) flow cytometry analysis of the surface markers of PDLSCs; (e–i) flow cytometry analysis of the surface markers of BMSCs.

We used flow cytometry to detect the characteristic MSC surface markers. The result showed that PDLSC-derived cells were positive for the MSC markers STRO-1, CD146, and CD90 and barely expressed the hematopoietic marker CD45 (Figures 1(a)–1(d)), which indicates that PDLSCs were successfully obtained. Flow cytometry analysis showed that BMSCs had high expression levels of MSC markers, such as CD44, CD90, and CD29, and low expression of CD11b/c and CD45 (Figures 1(e)–1(i)).

### 3.2. Characteristics of PDLSC-Derived Exosomes

**Figure 2:**
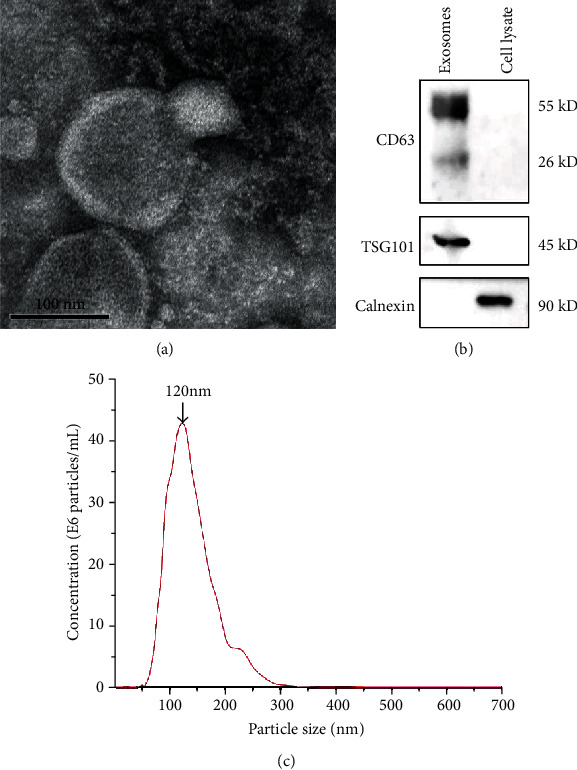
Characterization of exosomes derived from hPDLSCs. (a) TEM images confirmed the presence of exosomes, seen as cup-shaped vesicles. Scale bar: 100 nm. (b) Western blot analysis of the exosome-specific markers (TSG101, CD63) and endoplasmic reticulum protein (calnexin). Cell lysate of PDLSCs was used as control. (c) The particle size distribution and concentration of exosomes were measured by NTA.

The ultrastructure of exosomes was detected by TEM, which showed a typical cup-shaped morphology (Figure 2(a)). The western blot results showed that PDLSC-derived exosomes expressed exosomal surface markers (TSG101, CD63) while the nonexosomal marker (Calnexin) was hardly expressed (Figure 2(b)). In addition, NTA results showed that the average size of exosomes was 120 nm and the concentration of exosomes was 4.3 × 10^7^ particles/mL (Figure 2(c)).

### 3.3. Internalization of Exosomes by BMSCs

**Figure 3:**
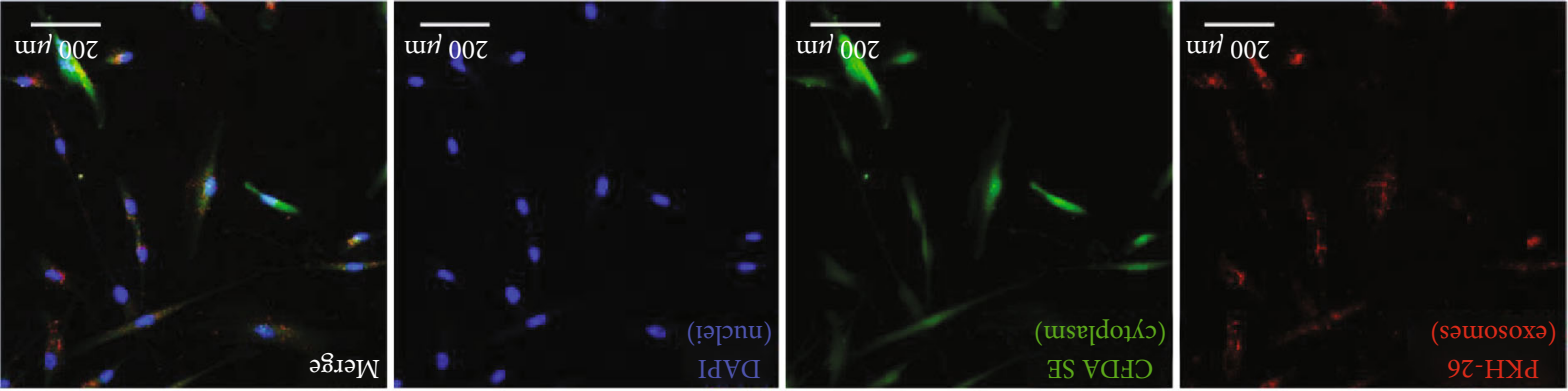
Internalization of exosomes by rBMSCs. PKH-26-labeled exosomes (red) were accumulated in the cytoplasm of rBMSCs after 6 h coincubation. The nuclei of rBMSCs were stained with DAPI (blue). The cytoplasm of rBMSCs was stained with CFDA-SE (green). Scale bar: 200 *μ*m.

To investigate whether BMSCs can internalize exosomes, exosomes were stained with PKH-26 and incubated with BMSCs for 6 hours. Fluorescence microscopy analysis (Figure 3) showed that a large number of exosomes (red dots) were internalized in the cytoplasm (green) of BMSCs after only 6 h of incubation.

### 3.4. The Effects of Exosomes from hPDLSCs during Different Periods of Osteogenic Induction on the Osteogenic Differentiation of BMSCs

**Figure 4:**
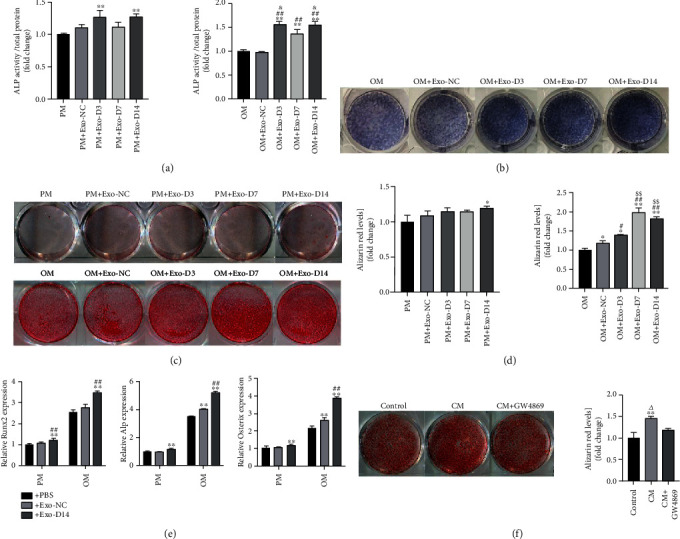
hPDLSC-derived exosomes enhanced the osteogenic differentiation of rBMSCs. (a) Alkaline phosphatase (ALP) activity normalized against the total protein content. *N* = 3/group. (b) ALP assays of BMSCs after incubation with 50 *μ*g/mL exosomes from PDLSCs after 0, 3, 7, and 14 days of osteoinduction at day 7. (c) Alizarin red staining (ARS) of BMSCs after incubation with 50 *μ*g/mL exosomes from PDLSCs after 0, 3, 7, and 14 days of osteoinduction at day 14. (d) Semiquantitive analysis of alizarin red staining (ARS). *N* = 3/group. (e) Expression of RUNX2, ALP, and Osterix in rBMSCs cultured with or without exosomes at 7 days. *N* = 3/group. (f) Alizarin red staining (ARS) of BMSCs after being cocultured with conditioned medium (CM) of PDLSCs treated with or without GW4869. *N* = 3/group. PM: proliferation medium; OM: osteogenic induction medium; ^∗^*p* < 0.05 compared with the control group (PM/OM/control group); ^∗∗^*p* < 0.01 compared with the control group; ^#^*p* < 0.05 compared with the Exo-NC group; ^##^*p* < 0.01 compared with the Exo-NC group; ^$^*p* < 0.05 compared with the Exo-D3 group; ^&^*p* < 0.05 compared with the Exo-D7 group. ^△^*p* < 0.05 compared with the GW4869 group.

To investigate the osteogenic inductive property of different exosomes, BMSCs were exposed to 50 *μ*g/mL exosomes derived from undifferentiated PDLSCs (Exos_NC) and osteogenically differentiated PDLSCs of different time points (Exos_D3, Exos_D7, Exos_D14) in either PM or OM. An equal volume of PBS was added to the control group. The ALP activity analysis showed that the exosomes derived from osteogenically induced PDLSC of different time points could significantly enhance the ALP activity of BMSCs in OM (*p* < 0.01) and the ALP activity under stimulation by Exos_D3 and Exos_D14 was dramatically higher than that by exosomes of other time spans (*p* < 0.05). Exosomes (Exos_NC) derived from nonosteogenic-induced PDLSCs were not able to enhance the ALP activity of BMSCs either in PM or OM (*p* > 0.05) (Figure 4(a)). The ALP staining under stimulation by exosomes derived from osteogenically induced PDLSC (Exos_D3, Exos_D7, and Exos_D14) was significantly enhanced in OM (Figure 4(b)). ARS staining showed an increase in calcium nodule formation in the Exos_14 group in PM and in the Exos_D7 and Exos_14 groups in OM (*p* < 0.05) (Figures 4(c) and 4(d)). The above results indicated that Exos_D14 could effectively promote the osteogenic differentiation of BMSCs. Consequently, we selected exosomes derived from the PDLSCs that were osteogenically induced for 14 days in the following experiments. qPCR was performed 7 days after treatment to investigate the expression of osteogenesis-related genes. Compared with BMSCs treated with Exos_NC, RUNX2 was upregulated in BMSCs treated with Exos_D14 under PM and OM conditions (*p* < 0.01) (Figure 4(e)). Compared with Exos_NC, Exos_D14 can significantly enhance ALP and Osterix expression in BMSCs when cultured in OM (*p* < 0.01) (Figure 4(e)). We prevent the formation of exosomes by treating the PDLSCs with the neutral sphingomyelinase (N-SMase) inhibitor GW4869. After 14 days, ARS staining showed a decrease in calcium nodule formation in BMSCs cultured in conditional medium with exosome blocked (*p* < 0.05) (Figure 4(f)), which further emphasized the role of exosomes in promoting osteogenic differentiation.

### 3.5. miRNA Profiles of Exosomes Derived from Osteogenically Differentiated PDLSCs and Undifferentiated PDLSCs Are Altered

**Figure 5:**
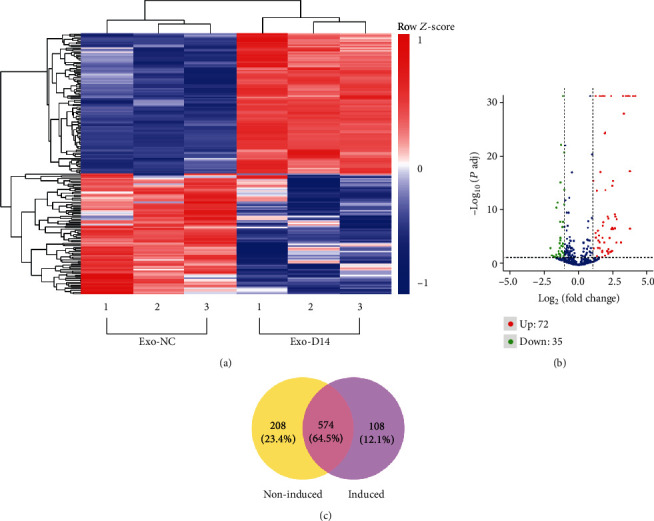
The exosomal miRNA differential expression profiles in undifferentiated and osteogenic differentiated PDLSCs: (a) the heat map of differentially expressed exosomal miRNAs; (b) the volcano plot of differentially expressed exosomal miRNAs; (c) the Venn graph showed that among differentially expressed miRNAs, 574 common miRNAs and 208 specific miRNAs in the nonosteogenic-induced group and 108 specific miRNAs in the induced group were identified.

We analyzed differentially expressed miRNAs in exosomes derived from undifferentiated and osteogenic differentiated PDLSCs by RNA sequencing. A total of 872 known mature miRNAs and 790 known pre-miRNAs were expressed in PDLSC-derived exosomes. 18 novel mature miRNAs and 19 novel pre-miRNAs were detected as well. As shown in Figure 5(a), the abscissa of the heat map represents sample clusters and the ordinate represents gene clusters. 72 miRNAs were upregulated, and 35 miRNAs were downregulated in exosomes derived from osteogenic differentiated PDLSCs compared with exosomes derived from undifferentiated PDLSCs (FC > 2, corrected *p* value < 0.05), as represented by the volcano plot (Figure 5(b)). Meanwhile, we identified 574 common miRNAs and 208 specific miRNAs in the nonosteogenic-induced group and 108 specific miRNAs in the osteogenic-induced group. The result was presented in the Venn diagram in Figure 5(c).

### 3.6. qPCR Verification of miRNA Expression

**Figure 6:**
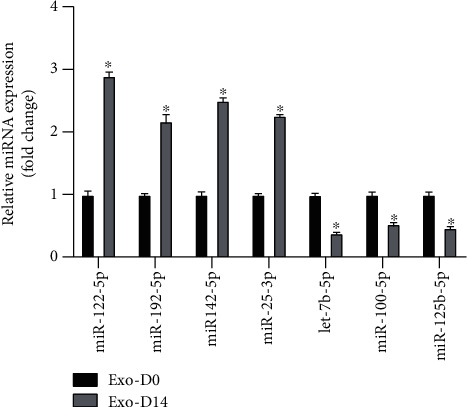
Validation of RNA sequencing data by qRT-PCR. miRNA expression was normalized to U6 expression. *N* = 3/group, ^∗^*p* < 0.05.

We chose seven miRNAs (miR-122-5p, miR-142-5p, miR-25-3p, miR-100-5p, miR-192-5p, miR-125b-5p, let-7b-5p) to verify the results of RNA-sequencing analyses using RT-qPCR. Compared with exosomes derived from undifferentiated PDLSCs, the expression of four miRNAs (miR-122-5p, miR-142-5p, miR-25-3p, miR-192-5p) in exosomes derived from osteogenically differentiated PDLSCs was significantly increased (*p* < 0.05), while the expression of miR-125b-5p, let-7b-5p, and miR-100-5p was decreased (*p* < 0.05) (Figure 6). The qPCR results were consistent with the conclusion obtained by RNA sequencing.

### 3.7. Pathway Analysis and GO Analysis of Exosomal miRNAs

**Figure 7:**
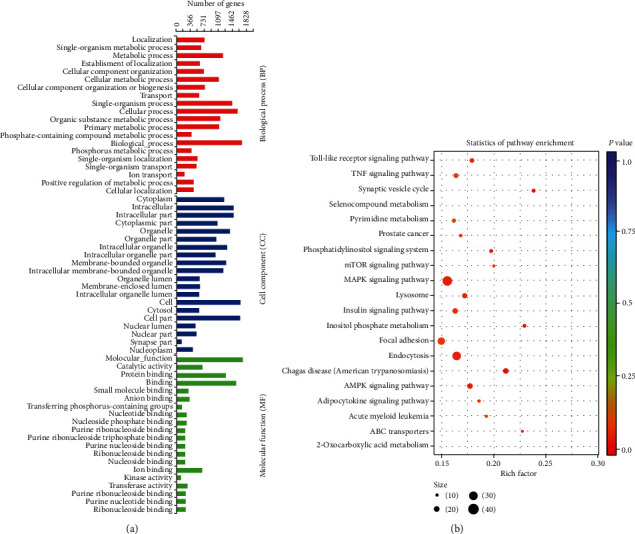
GO analyses and KEGG pathway analysis: (a) enrichment map of GO analyses—biological process, cellular component, and molecular function; (b) enrichment map of KEGG pathway analysis.

Exosomal miRNAs can affect the osteogenic differentiation process by targeting related genes that regulate corresponding signaling pathways, which may involve in the differentiation of stem cells. Therefore, we performed GO enrichment analyses to explore the potential function of differentially expressed miRNAs. According to GO analysis results, we found that functions such as catalytic activity (*p* value = 9.54*E*-12), protein binding (*p* value = 1.48*E*-11), metabolic process (*p* value = 8.32*E*-10), transport (*p* value = 2.75*E*-09), and phosphate-containing compound metabolic process (*p* value = 4.96*E*-08) are mainly affected by differentially expressed miRNAs (Figure 7(a)). The KEGG pathway analysis revealed that the target genes of the differentially expressed miRNAs (FC > 2, *p* < 0.05) are mainly related to processes such as 2-oxocarboxylic acid metabolism (*p* value = 0.01), adipocytokine signaling pathway (*p* value = 0.03), AMPK signaling pathway (*p* value = 0.03), insulin signaling pathway (*p* value = 0.04), and MAPK signaling pathway (*p* value = 0.04), as shown in Figure 7(b).

### 3.8. miRNA-mRNA Network Analysis

**Figure 8:**
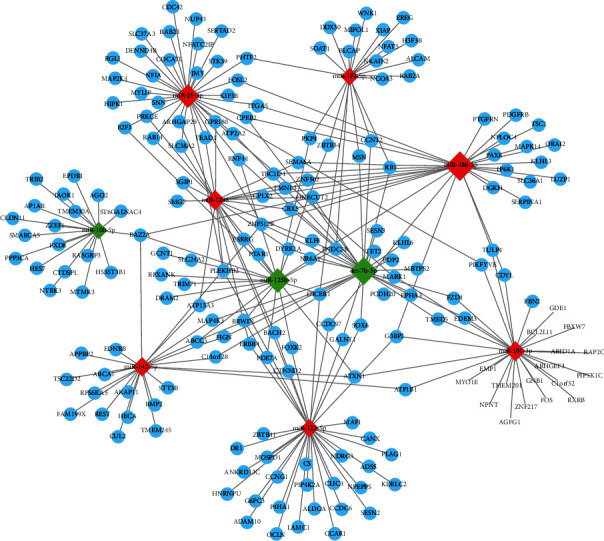
miRNA-mRNA network. The relationship between differentially expressed miRNAs and their target genes.

To explore the link between differentially expressed miRNAs and their target genes, we predicted the target genes of ten miRNAs with the most obvious differential expression (miR-122-5p, miR-142-5p, miR-25-3p, miR-100-5p, miR-192-5p, miR-125b-5p, let-7b-5p, miR-486-3p, miR-101-3p, miR-1246) and conducted the miRNA-mRNA network. As shown in Figure 8, the red diamonds represent upregulated miRNAs and the green diamonds represent downregulated miRNAs. The size of the diamond reflects the number of target proteins corresponding to each miRNA.

## 4. Discussion

Increasing studies have shown that MSC-derived exosomes have therapeutic effects on wound healing and tissue repair in various physiological systems [18, 33–35]. Periodontal ligament stem cells (PDLSCs) [36] have been reported as reliable sources of exosomes. Compared with other MSCs, hPDLSCs can be promising parent cells due to their easier accessibility, lower donor site morbidity, and the lowest invasive surgery requirements. hPDLSC was reported to show better regeneration capability and multipotency over DPSCs and DFPC [5]. They have great potential for many clinical applications. Most importantly, they are discarded if not used [37]. Exosomes derived from PDLSCs have been reported to exhibit the ability for reparation, such as promoting angiogenesis [38], regulating osteoblasts [39], alleviating pathology of neurological diseases [11], and alleviating inflammatory microenvironment [40]. Pizzicannella et al. [14] have proven that the addition of PDLSC-derived exosomes to 3D collagen membrane and polyethyleneimine scaffold can promote bone regeneration of skull defects in vivo.

Li et al. [18] reviewed that MSC-derived exosomes can promote osteogenesis through four major mechanisms, and the effect on promoting osteogenic differentiation of MSCs is considered one of the most critical mechanisms when MSC-derived exosomes are applied for bone tissue engineering. It has been indicated that the coculture of PDLSCs and BMSCs can promote the osteogenic differentiation potential and capability of ECM formation of BMSCs, and the exosomes derived from PDLSCs were speculated to be important mediators of intercellular communication [41].

In this study, we coculture BMSCs with exosomes derived from undifferentiated PDLSCs and osteogenically induced hPDLSCs to explore their ability to promote the osteogenesis of BMSCs *in vitro*. Our research indicates that the osteogenic induction of the hPDLSCs significantly enhanced the osteoinductive capacity of their exosomes. The ability to induce osteogenesis increases as the induction time increases. The exosomes derived from PDLSCs in the late stages of osteogenic differentiation showed the most significant effect in increasing ALP activity. The expression of RUNX-2, ALP, and Osterix was highly upregulated in BMSCs treated with Exo-D14. However, exosomes alone could promote but not sufficiently induce the complete in vitro osteoblastogenesis of rBMSCs. Importantly, we found that the calcium nodule formation in BMSCs was decreased when we blocked exosome secretion by GW4869, which further demonstrated the osteogenic effects of the exosomes. The contents of exosomes, such as nucleic acids (DNA, mRNA, miRNA, lncRNA, etc.), are of importance for the biological effects mediated by exosomes [42]. It has been reported that miRNAs are highly enriched in extracellular vesicles (EVs) [43], and the miRNAs in exosomes may vary with culture conditions. Liu et al. [44] reported different miR-155 abundance in exosomes secreted by lean ATMs and obese ATMs, which may contribute to varying abilities in modulating insulin sensitivity. Alteration of the exosomal miRNA expression was also reported in ADSCs during osteogenic induction [17]. Therefore, we hypothesize that exosomal miRNAs may be partially responsible for the observed effects.

Our study revealed differential expression of 108 exosomal miRNAs (72 upregulated and 35 downregulated) derived from osteogenically differentiated PDLSCs, among which many of the most changed miRNAs have been reported to be osteogenic-related miRNAs. miR-122-5p, miR-142-5p, miR-25-3p, miR-192-5p, etc. which have been reported as positive regulators of osteogenesis were upregulated in exosomes derived from osteogenic differentiated PDLSCs. BMSC-derived exosomes carrying miR-122-5p have been reported to promote the proliferation of osteoblasts in osteonecrosis of the femoral head [45]. lncRNA BLACAT1 targeting miR-142-5p can promote proliferation and osteogenic differentiation of BMSCs [46]. BMSC-secreted exosomal miR-192-5p can delay the event of the inflammatory response in rheumatoid arthritis [47]. Interestingly, miR-100-5p was dramatically decreased in exosomes derived from osteogenic differentiated PDLSCs. Previous reports have suggested that miR-100 acts as an essential endogenous negative regulator of BMP-induced osteoblast differentiation [48]. miR-145-3p and miR-143-5p, which were reported to be negative regulators of osteogenesis, were downregulated in exosomes derived from osteogenic differentiated PDLSCs. miR-143-5p was found to be downregulated in osteoporosis caused by impaired Wnt signaling [49]. Taken together, the regulation of the expression of osteogenic-related miRNAs, such as lower expression of exosomal miR-100-5p, miR-143-5p, and miR-145-3p and higher expression of exosomal miR-122-5p, miR-25-3p, and miR-142-5p from the osteogenic differentiated PDLSCs, may contribute to the promotion of osteogenic induction.

Furthermore, the GO analyses showed that a majority of the differentially expressed exosomal miRNAs involved in important molecular mechanism during osteogenic differentiation such as localization, metabolic process, cellular component organization or biogenesis, molecular function, catalytic activity, and protein binding. KEGG showed that the target genes are mainly enriched in signaling pathways regulating osteogenic differentiation, including the MAPK signaling pathway, AMPK signaling pathway, insulin signaling pathway, FOXO signaling pathway, and PI3K-Akt signaling pathway. It has been reported that inhibition activation of AMPK could significantly decrease the expression of OCN, Runx2, and ALP following BMP2-enhanced mineralization of osteoblasts [50]. Melatonin promotes the BMP9-induced osteogenic differentiation of mesenchymal stem cells by activating the AMPK/*β*-catenin signaling pathway [51].

Therefore, it is suggested that miRNAs may affect osteogenic differentiation by regulating their downstream target genes and pathways. For example, miR-122-5p, with the greatest fold change, was confirmed to be significantly upregulated in Exos-D14 by qPCR. Pyruvate kinase muscle isoenzyme 2 (PKM2) is predicted to be a highly likely target of miR-122-5p, and a study of renal cancer found that overexpressed miR-122-5p can negatively regulate PKM2 [45], which is consistent with our prediction. The previous study has demonstrated that PKM2 played an important role in the oxidative phosphorylation process and reported about the interaction of PKM2, AMPK, and *β*-catenin complex [44]. PKM2 can decrease active *β*-catenin and inhibited the transport of active *β*-catenin into the nucleus [52]. The inhibition of PKM2 can promote the osteogenic differentiation of BMSCs [53].

In our study, we found that 50 *μ*g/mL PDLSC-derived EVs can cause osteogenic changes in BMSCs. Other exosomes derived from MSCs showed a similar effect, but the dose that can cause osteogenic changes varies with the type of stem cells. Li et al. [18] demonstrated that the ability to promote BMSC osteogenic differentiation raised in exosomes derived from osteogenically induced hASCs and 25 *μ*g/mL osteogenically induced hASC-exosomes was sufficient to promote the osteogenic differentiation of hBMSCs in OM but not in FM. 100 *μ*g/mL iPSC-MSC-exosomes have been reported to promote the osteogenesis of BMSCs *in vitro* [54]. The difference in the effective dose may be due to the difference in the contents of exosomes. A recent study showed that exosomes secreted by hASCs and hBMSCs are enriched in distinctive miRNA and tRNA species, which indicates that the tissue origin might influence the contents and function of exosomes [55].

MSC-derived exosomes have been promising future perspectives for the development of cell-free therapies in human patients and have several potential advantages: (1) MSC-derived exosomes are normally hypoimmunogenic [56]; (2) use of exosomes avoids the safety concerns associated with stem cell transplantation and is less technically challenging to prepare and deliver [57]; and (3) exosomes are small enough to cross the barriers (blood-brain barrier, capillaries) [58]. The disadvantage is that they may not be able to produce more in vivo after they are transplanted. Then, questions arise about the efficacy and therapeutic dose of vesicles. In this *in vitro* study, PDLSC-derived exosomes have a similar therapeutic dose compared with other promising MSCs. It is necessary to apply exosomes to animals to further verify their bone formation effect and therapeutic dosage in our future work. If PDLSC-derived exosomes show good osteogenic effects *in vivo*, they may serve as excellent “inducing factors” in bone engineering and aid in repairing clinical bone defects in the future. In addition, exosomal miRNA may play a crucial role in promoting osteogenic differentiation of BMSCs and enhancing bone regeneration. However, the detailed mechanism needs to be further explored. This study can lay the foundation for understanding the mechanism of exosome-mediated promotion of BMSC osteogenic differentiation.

## 5. Conclusions

In conclusion, to the best of our knowledge, this is the first study concerning the ability of PDLSC-derived exosomes in promoting osteogenesis and the expression of their exosomal miRNAs. We have successfully identified several differentially expressed exosomal miRNAs from hPDLSCs under osteogenic induction and conducted relative bioinformatics analysis. These exosomal miRNAs may be crucial in bone regeneration. This study provided a new basis for research on bone regeneration and demonstrated the potential of PDLSC-derived exosomes in the development of novel therapeutic strategies.

## Figures and Tables

**Table 1:** List of gene primers.

Gene	Forward sequence	Reverse sequence
ALP	AGGGTGGGTTTCTCTCTTGG	AGAGAAGGGGTAGGGGAGAG
RUNX2	GGGACCGACACAGCCATATA	TCTTAGGGTCTCGGAGGGAA
Osterix	AGAGATCTGAGCTGGGTAGAGG	AAGAGAGCCTGGCAAGAGG
GAPDH	GGCACAGTCAAGGCTGAGAATG	ATGGTGGTGAAGACGCCAGTA
U6	GCAAATTCGTGAAGCGTTCCATA	AACGAGACGACGACAGAC
miR-122-5p	CCTGGAGTGTGACAATGGTGTTTG	
miR-142-5p	CCGCGCATAAAGTAGAAAGCACTAC	
miR-25-3p	CATTGCACTTGTCTCGGTCTGA	
miR-100-5p	CCAACCCGTAGATCCGAACTTGTG	
miR-192-5p	CGCTGACCTATGAATTGACAGCC	
let-7b-5p	CGCTGAGGTAGTAGGTTGTGTGGTT	
miR-125b-5p	CCTCCCTGAGACCCTAACTTGTGA	

## Data Availability

The data used to support the findings of this study are available from the corresponding author upon request.
